# The application of motion capture technology in a clinical evaluation and a therapy for people with autism spectrum disorder

**DOI:** 10.1192/j.eurpsy.2021.1843

**Published:** 2021-08-13

**Authors:** S. Baasansuren, D. Kaźmierczak, M. Świątko, K. Krysta

**Affiliations:** 1 Students` Scientific Association, Department Of Ophtalmology, Medical University of Silesia, Katowice, Poland; 2 Students` Scientific Association, Department Of Anesthesiology And Intensive Care, Medical University of Silesia in Katowice, Katowice, Poland; 3 Department Of Rehabilitation Psychiatry, Medical University of Silesia, Katowice, Poland

**Keywords:** autism spectrum disorder, Motion Capture, facial expression

## Abstract

**Introduction:**

Autism spectrum disorder (ASD) encopasses disorders with incompletely known etiology. Facial expression of people with ASD does not often reflect their emotions adequately or are strongly limited. In addition, they have a problem with joint attention. The symptoms of autism spectrum disorder are very various and have different severity that can change over time.There are still no objective methods for estimating these symptoms, which creates a huge diagnostic and clinical problem. Motion Capture technology makes the possibility of this objective assessment of the severity of initial symptoms, their change over time, as well as specificity for people with ASD.

**Objectives:**

To assess the application of Motion Capture technology in a clinical evaluation and a therapy for people with ASD.

**Methods:**

We analyzed literature related to the topic available at medical bases: PubMed, ResearchGate and Google Scholar. The articles which were included had been published after 2000 and have an English or Polish abstract.

**Results:**

We included 2 trials involving 81 participants (children and adolescents): 1 trial reported on quantifying the social symptoms of autism and 1 trial on differences of facial expressions in people with and without ASD.

**Conclusions:**

This capture of motions and the analysis of specific movements of people with autism spectrum disorder might be very useful in clinical practice, scientific research, therapy and also in creation of functioning systems at homes, schools and kindergartens. Thanks to this, people with ASD will be able to function better in society.

**Disclosure:**

No significant relationships.

